# Malaria in Pregnancy Is a Predictor of Infant Haemoglobin Concentrations during the First Year of Life in Benin, West Africa

**DOI:** 10.1371/journal.pone.0129510

**Published:** 2015-06-08

**Authors:** Manfred Accrombessi, Smaïla Ouédraogo, Gino Cédric Agbota, Raquel Gonzalez, Achille Massougbodji, Clara Menéndez, Michel Cot

**Affiliations:** 1 Institut de Recherche pour le Développement (IRD), UMR216-Mère et enfant face aux infections tropicales, Paris, France; 2 PRES Paris Sorbonne Cité, Université Paris Descartes, Paris, France; 3 Faculté de Médecine de Cotonou, Université d’Abomey-Calavi, Cotonou, Benin; 4 Unité de Formation et de Recherche en Sciences de la Santé, Université de Ouagadougou, Ouagadougou, Burkina Faso; 5 ISGlobal, Barcelona Centre for International Health Research (CRESIB), Hospital Clínic-Universitat de Barcelona, Barcelona, Spain; 6 Manhiça Health Research Center (CISM), Manhiça, Mozambique; Shoklo Malaria Research Unit Mahidol University and University of Oxford, THAILAND

## Abstract

**Background:**

Anaemia is an increasingly recognized health problem in Africa, particularly in infants and pregnant women. Although malaria is known to be the main risk factor of anaemia in both groups, the consequences of maternal factors, particularly malaria in pregnancy (MiP), on infant haemoglobin (Hb) concentrations during the first months of life are still unclear.

**Methods:**

We followed-up a cohort of 1005 Beninese pregnant women from the beginning of pregnancy until delivery. A subsample composed of the first 400 offspring of these women were selected at birth and followed until the first year of life. Placental histology and blood smear at 1^st^ clinical antenatal visit (ANC), 2^nd^ ANC and delivery were used to assess malaria during pregnancy. Infant Hb concentrations were measured at birth, 6, 9 and 12 months of age. A mixed multi-level model was used to assess the association between MiP and infant Hb variations during the first 12 months of life.

**Results:**

Placental malaria (difference mean [dm] = - 2.8 g/L, 95% CI [-5.3, -0.3], P = 0.03) and maternal peripheral parasitaemia at delivery (dm = - 4.6 g/L, 95% CI [-7.9, -1.3], P = 0.007) were the main maternal factors significantly associated with infant Hb concentrations during the first year of life. Poor maternal nutritional status and malaria infection during infancy were also significantly associated with a decrease in infant Hb.

**Conclusion:**

Antimalarial control and nutritional interventions before and during pregnancy should be reinforced to reduce specifically the incidence of infant anaemia, particularly in Sub-Saharan countries.

## Introduction

The World Health Organization (WHO) has estimated that over 50% of children less than 4 years are anaemic in developing countries [1]. Iron deficiency is probably the most important cause but other nutrient deficiencies, malaria, intestinal parasitic infections, and chronic infections such as HIV also play an important role in low income countries. Malaria in pregnancy (MiP), a commonplace disease in these areas [2], is the main risk factor for anaemia during gestation, with the most important negative impact on maternal haemoglobin (Hb) observed in primigravidae [3].

In Africa, very few studies have focused on the effects of maternal factors during pregnancy on postnatal child’s health. Although the risk factors of anaemia in childhood are fairly well known [4–7], the influence of gestational factors such as maternal haematological parameters, nutritional status and history of infections occurring during gestation on the infants’ Hb concentrations during the first year of life have been poorly explored.

On the occasion of a multi-center trial of Intermittent Preventive Treatment in Pregnancy (IPTp) comparing sulfadoxine-pyrimethamine and mefloquine (MiPPAD study “Malaria in Pregnancy Preventive Alternative Drugs”) [8], we had the opportunity to follow a cohort of 1005 pregnant women with the objective to study anaemia during pregnancy and its consequences, APEC study (Anaemia in Pregnancy: Etiology and consequences). The first 400 children of these women were followed-up to investigate the impact of different maternal factors in pregnancy especially MiP on infant Hb variations during the first year of life in southern Benin.

## Materials and Methods

### Study design

Prospective cohort study, set-up between January 2010 and May 2012 to follow pregnant women from the first antenatal clinical visit (1^st^ ANC) to delivery in the framework of the MiPPAD clinical trial. The first 400 infants to be delivered from these women, were enrolled from January 2010 until June 2011 and followed throughout the first year of life.

### Study site and population

The study was conducted in two maternity clinics (Attogon and Sékou) in the district of Allada, a semi-rural area located 50 km north of Cotonou and the economic capital of Benin. Allada district is characterized by a subtropical climate and malaria is hyperendemic with an average of 20.5 infected anopheles bites/person/year [9]. *Plasmodium falciparum* is the predominant species transmitted (97%).

The study population was composed of HIV—negative pregnant women and their children residing in the district of Allada. Twin pregnancies, pregnancies complicated by stillbirth or fetal abnormalities have been excluded. IPTp was administered according to the clinical trial protocol and the details are presented elsewhere [8]. Briefly, two doses of IPTp (1,500/75 mg of sulfadoxine-pyrimethamine or 15 mg/kg of mefloquine per dose) were administered at ANC visits. The second dose of IPTp was given at least 1 month apart from the first dose. Clinical malaria episodes were treated with oral quinine or arthemether-lumefantrine in first and subsequent trimesters, respectively, for uncomplicated malaria, and with parenteral quinine in case of severe malaria. Children with clinical malaria were also treated with arthemether-lumefantrine according to the national malaria control program guidelines and in case of severe malaria, they were referred to the district hospital. Each woman received a long-lasting insecticide-treated net that was replaced in case of damage or loss during the follow-up. They were also systematically given 600 mg of mebendazole at 1^st^ ANC to be taken at home (100 mg twice day for 3 days) according to the guidelines of the Beninese Ministry of Health. In addition, supplements of oral ferrous sulfate (200 mg/day) and folic acid (5 mg/day) were given to the pregnant women to be taken at home. Pregnant women with a Hb concentration below 110g/L were treated according to the severity of anaemia (i.e., 200 mg oral ferrous sulfate twice day for mild and moderate anaemia (Hb level between 70 and 110 g/L) and referred to the district tertiary hospital in case of severe anaemia (Hb < 70 g/L)). Mild or moderate infant anaemia was treated with 10 mg/kg/day of iron syrup for two months. Children with severe anaemia were also referred to the district tertiary hospital for blood transfusion. Infant with positive stool test were treated with mebendazole syrup (100 mg twice day for 3 days). All the medications prescribed to the women and the children during their participation in the study were free of charge.

### Study procedures

#### Clinical data collection

After obtaining informed consent, we collected sociodemographic and socioeconomic characteristics of participants at enrolment. At the 1^st^ ANC, women were examined and gestational age, middle upper arm circumference (MUAC), weight and height were recorded. This information, except for height, was also collected at 2^nd^ ANC and delivery. Gestational age was determined from fundal height measurement by bimanual palpation and following McDonald's rules [10]. Weight and height in pregnant women were respectively measured to the nearest 0.1 kg using an electronic scale (Seca corp., Hanover, MD) and to the nearest 0.1 cm by using a bodymeter device (Seca 206 Bodymeter; Seca corp.). These parameters were measured by two nurses, and the mean of two measurements was calculated.

**Fig 1:**
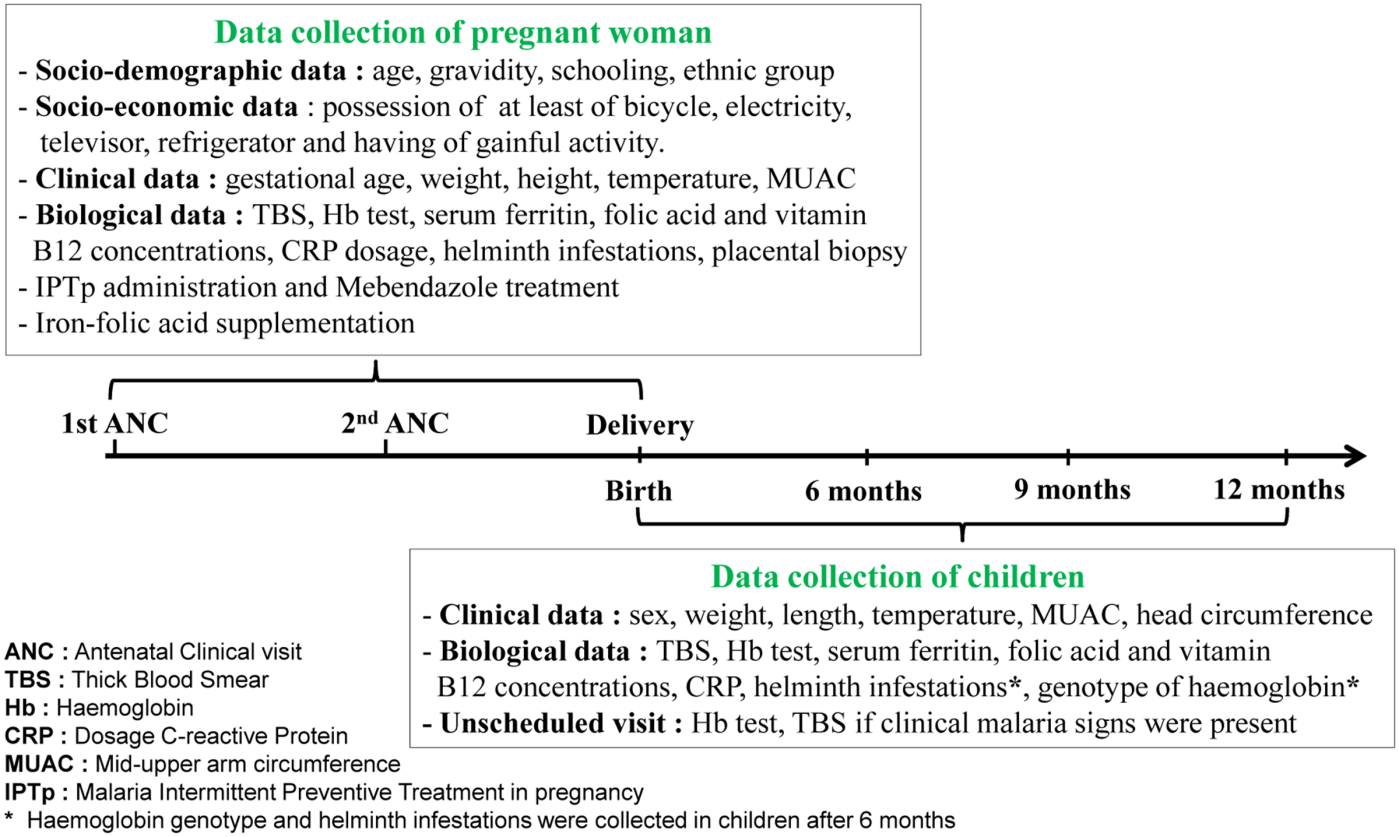
Study procedures. During follow-up, socio-demographic, economic, clinical and biological data were collected in mothers at 1^st^ antenatal clinical visit (ANC), 2^nd^ ANC and delivery. The same data were also recorded in infants at birth, 6, 9 and 12 months of life. Outside of scheduled visit, haemoglobin concentration and blood smear were performed when malaria signs were present.

At birth, newborn’s sex, weight, length, head circumference and axillary temperature were collected. Weight was measured using an electronic baby scale (SECA type 354) with a precision of 10 g and length was measured to the nearest 1 mm with a locally manufactured wooden measuring scale according to the criteria recommended by WHO. At the 6, 9 and 12 months scheduled visits, the history of fever within the previous 24 hours, malaria treatment or hospitalization since the last visit and use of insecticide-treated nets were recorded (Fig 1).

#### Blood and stool sample collection

At 1^st^ ANC, 2^nd^ ANC and delivery, 8 mL of mother’s venous blood was collected. The same volume was also collected on cord blood at birth and on infant's venous blood at 6, 9 and 12 months of life. All these samples were used to look for malaria parasitaemia, to determine C-reactive protein (CRP), micronutrient (serum ferritin, folic acid and vitamin B12) and Hb concentration and to genotype Hb. At delivery, samples (biopsy and impression smear) were collected from the placenta for parasitological evaluation. A container was also given to the woman to collect infant’s stools in search of intestinal helminths. On unscheduled visits, Hb dosages and thick blood smears were performed in infants with clinical signs of malaria (history of fever in the last 24 hours or temperature ≥ 37.5°C and pallor).

#### Laboratory methods

The Hb level was measured with a Hemo-Control photometer (EKF Diagnostics, Magdeburg, Germany) device. Daily calibration of the Hemo-Control device was performed by laboratory technicians. In addition, an external quality control was made by sending one of 10 consecutive samples to the Allada Central Hospital laboratory, where dosages were assessed using a hematology analyzer (Erma Laboratory, Tokyo, Japan). Hb genotypes were determined by alkaline electrophoresis on cellulose acetate (Helena laboratories, Beaumont, TX).

Serum ferritin, folic acid, and vitamin B12 concentrations were measured using a microparticle enzyme and fluorescence polarization immunoassay (AxSym Immuno-Assay Analyzer, Abbott Laboratories). CRP concentration was determined by rapid slide test (CRP Latex; Cypress Diagnostics Inc.) to correct the effect of inflammatory syndromes on ferritin concentrations.

The Determine (HIV1 and 2 kit; Abbott Laboratories) and Bioline (HIV1 and 2 3.0 kit; Bioline, Taunton, MA) rapid tests were used to detect HIV infections using a serial testing algorithm.

The Lambaréné technique was used to analyze peripheral malaria infection in blood smears. It consists of spreading a calibrated 10 μL amount of blood on a slide’s rectangular area of 1.8 cm² (1.8 x 1 cm). The slide was stained with Giemsa and read at a magnification of 1,000 × with an oil immersion lens. A multiplication factor was applied to the average parasitemia/field to determine the number of parasites/μL. The Lambaréné method detection threshold has been estimated to be 5 parasites/μL [11,12].

Placental biopsies (2.5 x 2.5 cm^3^), collected at delivery for histology assessment, were immediately put in 50 ml of 10% buffered formalin. It was then stored at 4°C in a refrigerator until the placental tissue was processed at the pathology department. The maximum delay before fixation was of 5 days. Placental malaria infection was defined as the presence of parasites with /without pigment or pigment confined to fibrin in the histological examination [13]. Placental histology was examined without knowledge of the peripheral blood smears results. In addition, an external quality control was made on 100% of positive slide and 10% of negative slide in reference laboratory to Barcelona Centre for International Health Research (CRESIB), Hospital Clínic-Universitat de Barcelona.

Infestations by helminths were assessed by using the Kato-Katz concentration method (Vestergaard Frandsen, Lausanne, Switzerland).

### Definitions

Anaemia. Gestational anaemia was defined as a Hb concentration below 110 g/L [14]. In children, it was defined by Hb level below 140 g/L at birth [15] and below 110 g/L from 6 months [14]. Severe, moderate, and mild anaemia were respectively defined as Hb concentrations less than 80 g/L, between 80 and 99 g/L, and between 100 and 109 g/L. Maternal anaemia was also assessed by number of anaemia episodes during pregnancy and in purpose pregnant women have been classified into 4 groups: (1) women not presenting any episode of anaemia during pregnancy (either at inclusion, at the second antenatal visit or at delivery); (2) women presenting a single episode; (3) women with two episodes and (4) pregnant women constantly anaemic (anaemia at each of the 3 blood assessments).

Iron status and iron deficiency anaemia (IDA). Iron deficiency (ID) was defined as a serum ferritin concentration less than 12 μg/L or as serum ferritin concentration of 12–70 μg/L in the context of inflammatory syndrome (CRP concentration ≥ 6 mg/mL) [16]. Anaemia associated to ID determined IDA.

Folic acid and vitamin B12 deficiencies. Folic acid and vitamin B12 deficiencies were respectively defined as a serum folic acid concentration less than 6 ng/mL and a vitamin B12 serum concentration less than 150 pg/mL [17].

Helminth infestations. Intestinal helminth infestations were diagnosed by the presence of intestinal helminth eggs in the stool sample. Eggs were counted as number of eggs per g of stool.

Estimation of pre-pregnancy body mass index (BMI). All pregnant women included in the study had a gestational age less than 28 weeks. From the end of the first trimester of gestation, it was estimated that pregnant women gained on average 1 kg per month until delivery [18]. We used the gestational age at inclusion to estimate approximately the weight that women were supposed to have gained since the beginning of the pregnancy. This amount was then subtracted from the weight on the day of inclusion to obtain a rough estimate of the weight before pregnancy. BMI was calculated as the weight in kilograms divided by the square of the height in meters (kg/m²) and we used WHO criteria to classify the women in normal BMI, thinness (severe, moderate, mild), overweight and obesity [19].

Socio-economic index of family. This index was obtained from a synthetic score given by the sum of the following binary variables: having gainful activity, electricity, television, refrigerator and at least a bicycle. The total score was graded between 0 and 5. Low, average, high socio-economic indexes were defined as synthetic score ≤ 1, between 2–3, and ≥ 4, respectively.

### Data management and statistical analysis

Data were double-entered into Microsoft Access 2003 database and analyzed with Stata 12.0 Software for Windows (Stata Corp, College Station, TX). Infant Hb concentration (g/L) was the main outcome variable in our study. Malaria infection during pregnancy (either placental infection or peripheral parasitaemia) was the main exposure variable. We used the following co-variables in mothers: age (years), ethnic group, socio-economic index, gravidity, gestational age (weeks), number of antenatal visits, BMI, maternal anaemia, iron, folic acid, and vitamin B12 deficiencies, marital status, education level. In the infants, we considered: age (months), sex, low birthweight ((LBW), weight < 2500 g), preterm birth (gestational age < 37 weeks), fever (temperature ≥ 37.5°C), inflammation syndrome, stunting (length-for-age z-score < -2SD), wasting (weight-for-length z-score < -2SD), malaria and helminth infections, sickle cell disease, serum ferritin, folic acid and vitamin B12 concentrations.

We first described the general characteristics of the women at delivery and their children at birth, 6, 9 and 12 months. The variations in the distribution of Hb levels between 0, 6, 9 and 12 months were assessed by a Kruskal-Wallis test. Proportions were compared with a Fisher exact test. At each time point (birth, 6, 9 and 12 months), infant Hb levels were compared with Student's t-test or Mann-Withney non-parametric test as appropriate.

Then, we assessed the effect of MiP on infant Hb at birth using multivariable linear regression. Afterwards, we used a longitudinal approach to take into account Hb variations over time and to estimate the impact of MiP on infant Hb throughout the first year of life. Assuming that successive Hb measurements in the same infant were correlated, the data presented a hierarchical two-level structure, where Hb measurements (level 1) were clustered within infants (level 2) [20]. Therefore, a linear mixed model with a random intercept was built as specified in the equation below:

$$\text{Haemoglobin}(\text{ij})={\text{β}}_{0}{}_{0}+\sum_{\text{q }=\text{ 1}}^{\text{n}}{\text{β}}_{\text{q0}}\;{\text{X}}_{\text{qj}}\;+\;{\text{U}}_{\text{0j}}\;+\;{\text{e}}_{\text{ij}}$$

where haemoglobin (ij) is the *i*th Hb measurement of infant j, *β*_00_ is the intercept, Xqj is the q explicative variable of infant j and *β*_q0_ its associated coefficient, u_0j_ is the random intercept corresponding to the infant-to-infant variation in Hb level [u_0j_ —N (0, π_00_)], and e_ij_ is the residual variation [e_ij_—N(0, σ²)]. Fixed effects parameters were estimated using the maximum likelihood method, and variance components were estimated using the restricted maximum likelihood method. To take into account the numerous physiological changes and the non linear evolution of Hb during the first 3 months of life, we excluded the first Hb measurement (at birth) from the hierarchical mixed model. Also, we adjusted our longitudinal analysis on the hematological parameters of the newborn such as iron or folic acid deficiencies at birth that could modify the levels of Hb during the first year of life. All variables with *P* values below 0.2 in univariate analysis and which were not in the causal pathway between placental malaria and infant Hb, were included in multivariate analyses. Manual backward selection procedure was performed and statistical significance was set at *P* < 0.05. For variables with more than two categories, *P* value of the global test is given.

### Ethics statement

This study was approved by the Ethics Committee of the Health Sciences Faculty of Cotonou in Benin. Before each inclusion, all participants involved in our study provided their written informed consent to participate in this study. The study was also explained in the local language to the participant, and her voluntary consent was obtained. In case the woman could not read, an impartial witness was involved in the process. Mothers were free to interrupt their participation at any time in the study.

## Results

### Study profile

**Fig 2:**
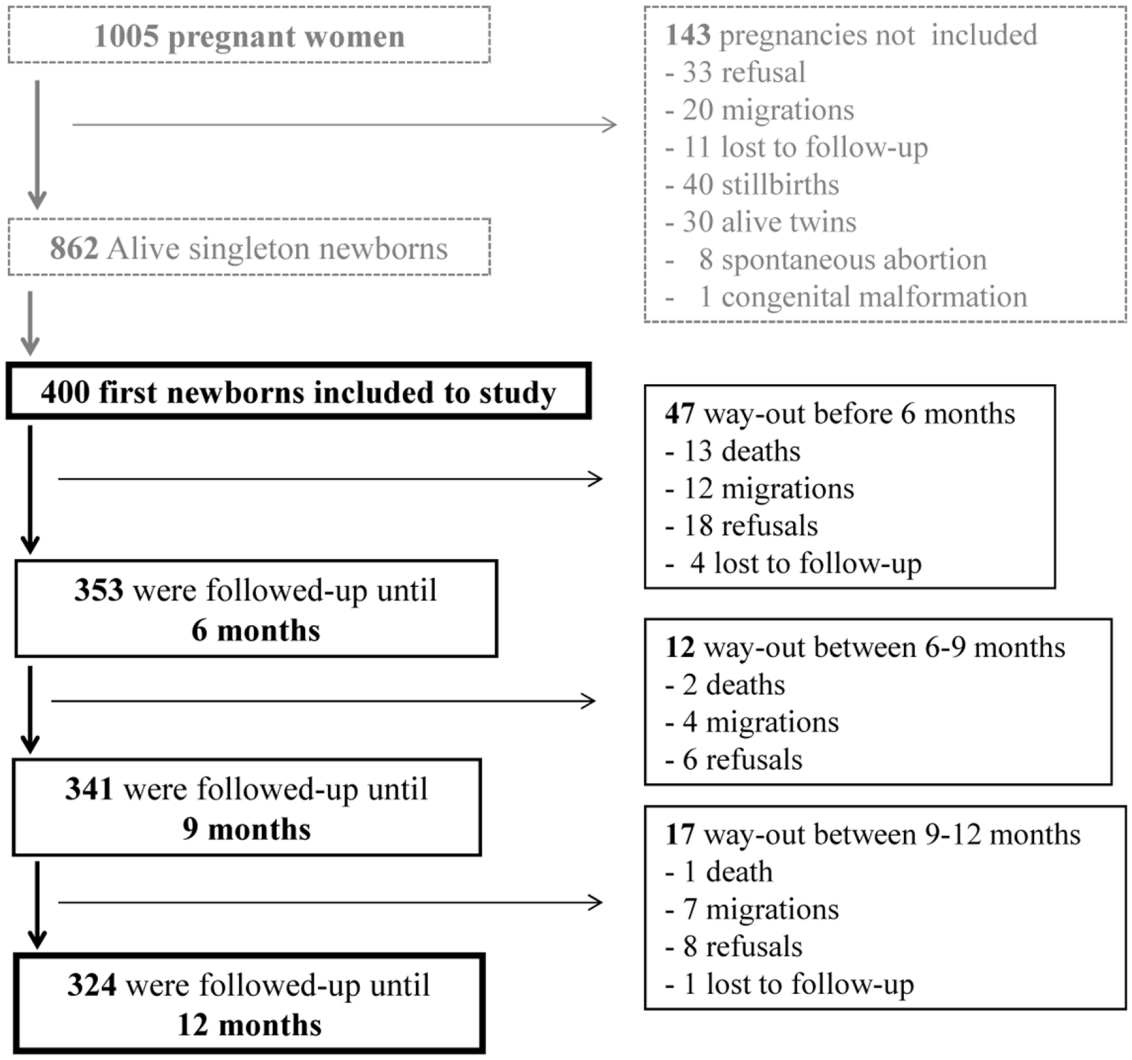
Flowchart diagram of follow-up. Infants who were absent more than 3 consecutive months, and not seen before their 12 months were considered as lost to follow-up. During the study, five infants (0.1%) were lost to follow-up and sixteen (0.4%) died. The main reasons of death were: acute respiratory infection (4), neonatal icterus (1), severe malaria (2), unknown disease (7), congenital biliary atresia (1). Among these deaths, only 1.2% (2/16) of infants have been bring to hospital by parents.

Between January 2010 and June 2011, 400 mother-infant pairs were enrolled in the study. The proportion of lost to follow-up during the whole study period was 1.2% (Fig 2). Hb was respectively assessed in 100% (400 of 400), 88.4% (312 of 353), 91.2% (311 of 341), and 97.5% (316 of 324) of infants at the time of birth, 6, 9 and 12 months.

### Characteristics of study population

Table 1 presents the general characteristics of pregnant women during study. Women’s mean age was 25.9 years (Standard deviation (SD) = 5.4) and 15.7% (63/400) of pregnant women were primigravidae. During the follow-up, 2.3% (9/400), 55.7% (223/400), 42% (168/400) had 2, 3 and ≥ 4 ANC visits, respectively. The mean of estimated pre-pregnancy BMI was 20.8 kg/m² (SD = 2.7) and 18.7% (75/400) of the mothers were underweight. Among those, 8% (32/400), 6.5% (26/400) and 4.3% (17/400) presented mild, moderate and severe thinness, respectively. The mean of gestational age at delivery was 39.8 weeks (SD = 1.8). The proportion of maternal anaemia decreased from 1^st^ ANC to delivery (72.5% to 50%) and 36.9% (145/392) of women were anaemic during all scheduled visits of pregnancy. Helminth infestations decreased progressively from the 1^st^ ANC (9.1%) to delivery (5.1%). The proportion of women with a positive blood smear decreased after IPTp administration (from 16% at 1^st^ ANC to 4.1% at 2^nd^ ANC), and increased again at delivery (10.8%). Eighty seven pregnant women (22.1%) had a placental malaria infection at histology evaluation. Infant families’ socio-economic level was low for 49.3% of the study population.

**Table 1:** General characteristics of pregnant women during study in district of Allada, Benin, 2010–2012.

Characteristics			ANC 1*		ANC 2*		Delivery
		No.	Mean or %	No.	Mean or %	No.	Mean or %
Duration between differents visits (days)		400	-		44.1 (±11.1)		79.7 (±12.7)
Age (years)	Mean	400	25.9 (±5.4)	-	-	-	-
Gravidity (%)	Multigravidae	337	84.3	-	-	-	-
IPTp group (%)	SP	138	35.5	-	-	-	-
	MQ	262	65.5	-	-	-	-
Estimated pre-pregnancy BMI (%)	BMI < 16.0	17	4.3	-	-	-	-
	16.0 ≤ BMI < 17.0	26	6.5	-	-	-	-
	17.0 ≤ BMI < 18.5	32	8.0	-	-	-	-
	18.5 ≤ BMI < 25.0	285	71.2	-	-	-	-
	25.0 ≤ BMI < 30.0	36	9.0	-	-	-	-
	BMI ≥ 30.0	4	1.0	-	-	-	-
Gestational age (weeks)	Mean	400	22.7 (±3.9)	392	29.2 (±3.7)	397	39.8 (±1.8)
	Preterm birth (%)	-	-	-	-	397	5.8%
Gestational anaemia (%)	Yes	400	72.5	392	67.1	400	50.0
Iron deficiency (%)	Yes	400	36.5	392	40.1	397	31.5
Folic acid deficiency (%)	Yes	400	33.7	392	17.1	397	43.5
Vitamin B12 deficiency (%)	Yes	400	5.3	392	3.6	397	9.3
Helminth infection (%)	Yes	395	9.1	387	8.8	355	5.1
Peripheral malaria infection (%)	Yes	400	16.0	392	4.1	400	10.8
Placental malaria infection^†^ (%)	None	-	-	-	-	307	77.9
	Past	-	-	-	-	45	11.4
	Chronic	-	-	-	-	33	8.4
	Active	-	-	-	-	9	2.3

**BMI**: Body mass index; **Preterm birth**: gestational age at delivery < 37 weeeks;

**IPTp**: Intermittent Preventive Treatment in pregnancy against malaria, **SP**: Sulfadoxine-Pyrimethamine, **MQ**: Mefloquine

* First and second dose of IPTp administrations; Standard deviation are in parentheses

^†^ Malaria infection detected in placenta by histology.

Table 2 shows the clinical and biological characteristics of the children during the first year of life. The newborns' mean Hb was 139 g/L (SD = 21) and 46.3% of them were anaemic. Newborns weighed on average 3033 g (SD = 420.4) and 9.1% (36/400) had a LBW. Only eight children (2.4%) had sickle cell disease (genotypes SS and SC). The distribution of Hb concentrations were significantly different between scheduled visits of infant (Kruskal-Wallis test, *P* < 0.01). The Hb level decreased from birth to 6 months and remained almost constant until 12 months (S1 Fig). Anaemia was common in the second half of infancy, exceeding 65% after 6 months and was significantly dissimilar between the different visits (46.3%, 66.9%, 70.1%, 64.6% at birth, 6, 9 and 12 months of life, respectively; Fisher exact test, *P* < 0.01). Over a third of the children presented with ID after 6 months. Malaria infection was common at all stages of follow-up, around 12%.

**Table 2:** Clinical and biological characteristics of children during the first year of life in district of Allada, Benin 2010–2012, N = 400.

Characteristics		At birth	At 6 months	At 9 months	At 12 months
		Mean or %	Mean or %	Mean or %	Mean or %
Sex (%)	Female	53	-	-	-
	Male	47	-	-	-
Weight (g)	Mean	3033 (±420.4)	7001 (±1001.1)	7705 (±1004.8)	8400 (±1089.1)
	LBW (%)	9.1	-	-	-
Length (cm)	Mean	48.9 (±2.43)	66.0 (±3.05)	69.6 (±3.12)	72.5 (±3.44)
MUAC (cm)	Mean	-	13.8 (±1.44)	14.1 (±2.15)	14.4 (±2.84)
Fever (%)		1.5	16.1	19.8	16.4
Wasting^†^ (%)		-	14.6	13.6	9.9
Stunting^‡^ (%)		-	14.3	13.6	16.7
Haemoglobin (g/L)	Mean	139.0 (±21.0)	102.1 (±14.8)	102.9 (±14.2)	103.7 (±14.9)
	Anaemia^¥^ (%)	46.3	66.9	70.1	64.6
	Mild (100–109)	4.0	31.4	34.7	36.1
	Moderate (80–99)	2.7	28.2	29.6	21.5
	Severe (< 80)	1.3	7.4	5.8	6.9
Iron deficiency (%)		0.5	25.7	38.9	46.2
Folic acid deficiency (%)		15.3	10.3	15.3	16.2
Vitamin B12 deficiency (%)		3.5	12.9	17.3	14.3
Malaria infection (%)		0	12.1	11.9	12.4
Helminth infection (%)		-	3.7	11.9	9.7
Inflammation (%)		1.3	24.5	28.8	27.9
Sickle cell disease (%)	(SS, SC)	-	2.4	-	-

**MUAC**: Mid-upper arm circumference; **LBW**: Low birthweight (weight < 2500 g); Standard deviation are in parentheses

^†^ Wasting: weight-for-length z-score < -2SD

^‡^ Stunting: length-for-age z-score < -2SD

^¥^ Anaemia: haemoglobin < 140 g/L (birth) and < 110 g/L (between 6 and 12 months).

### Effect of MiP on newborns' Hb concentrations at birth

Children born to malaria-infected mothers tended to present lower Hb concentrations than children born to non malaria-infected mothers at birth but the association was not significant (difference of mean Hb (dm) = −1.1 g/L, 95% CI [-3.9, 6.1], P = 0.30). After adjustment, newborn ferritin level was significantly associated with a decrease in newborns' Hb concentrations while first pregnancy, Aïzo ethnic group, newborn’s folic acid and vitamin B12 concentrations were significantly associated with an increase in Hb concentrations at birth (Table 3). Considering the timing of MiP, malaria infection occurring at 1^st^ ANC, 2^nd^ ANC and delivery were not statistically associated with newborn Hb at birth (Table 4).

**Table 3:** Factors associated with newborn haemoglobin concentration at birth in district of Allada, Benin 2010–2012, N = 392 (Univariate and multivariate linear regressions).

Factors		Crude mean haemoglobin difference (g/L)	95% CI	*P value*	Adjusted mean haemoglobin difference (g/L)	95% CI	*P value*
**Maternal factors**							
Placental malaria infection^†^		- 1.1	[-3.9, 6.1]	0.68	-	-	-
Number of anaemia episode during pregnancy	1	0.3	[-7.1, 7.6]		-	-	-
	2	- 2.6	[-9.6, 4.6]	0.35	-	-	-
	3	- 4.3	[-11.5, 2.6]		-	-	-
Maternal vitamin B12 deficiency at delivery		- 8.2	[-15.3, -1.1]	0.02	-	-	-
Maternal folic acid deficiency at delivery		- 3.4	[-7.6, 0.8]	0.11	-	-	-
Primigravidae		7.3	[1.6, 12.9]	0.01	8.5	[2.7, 14.2]	< 0.01
Aïzo ethnic group		5.1	[0.6, 9.5]	0.03	6.2	[1.7, 10.7]	< 0.01
IPTp group (Mefloquine)		- 3.2	[-7.6, 1.1]	0.14	-	-	-
**Child’s factors**							
Serum ferritin level of child at birth (μg/L)		- 2.6	[-5.2, 0.1]	0.05	- 4.4	[-7.2, -1.6]	< 0.01
Serum folic acid level of child at birth (ng/mL)		3.1	[0.2, 6.1]	0.04	3.9	[0.9, 6.9]	0.01
Serum vitamin B12 level of child at birth (pg/mL)		2.9	[-0.2, 6.2]	0.07	3.7	[0.4, 7.0]	0.03
Sex of child (male)		3.5	[-0.6, 7.7]	0.09	-	-	-

(-) Association was not significant in multivariate analysis; 95% CI: Confidence Interval to 95%

IPTp: Intermittent Preventive Treatment in pregnancy

^†^ Malaria infection detected in placenta by histology (included past, chronic and active infection).

**Table 4:** Effect of the timing of maternal peripheral parasitaemia during pregnancy on infant haemoglobin level (g/L) in district of Allada, Benin 2010–2012, by univariate analysis.

Malaria in pregnancy			Birth^†^			First year of life^‡^		
		No.	Mean haemoglobin difference (g/L)	95% CI	*P value*	Mean haemoglobin difference (g/L)	95% CI	*P value*
Peripheral malaria infection at first ANC								
	No*	336						
	Yes	64	- 4.8	[-10.4, 0.8]	0.09	- 0.5	[-3.7, 2.6]	0.74
Peripheral malaria infection at second ANC								
	No*	376						
	Yes	16	- 1.9	[-12.6, 8.6]	0.71	0.4	[-6.3, 7.1]	0.90
Peripheral malaria infection at delivery								
	No*	357						
	Yes	43	- 2.4	[-9.1, 4.2]	0.47	- 4.7	[-8.3, -1.1]	0.01

ANC: Antenatal clinical visit

^†^ Relationship assessed by linear regression

^‡^ Relationship assessed by multilevel linear regression (Hb measurement at birth excluded to analysis)

* Baseline category.

The relation between the number of maternal anaemia episodes during pregnancy and newborns’ Hb concentrations was not significant even if a lower Hb concentrations were observed in infants born to mothers with at least two episodes of anaemia during gestation (dm = −2.6 g/L, 95% CI [-9.6, 4.6] for newborns of women with 2 episodes of anaemia and dm = −4.3 g/L, 95% CI [-11.5, 2.6] for newborns of women with 3 episodes of anaemia, P = 0.35) (Table 3).

Newborns of mothers with more than 3 ANC visits tended to present lower Hb concentrations than those of mothers with less than 3 ANC visits, but the association was not statistically significant (dm = −1.01 g/L, 95% CI [-5.2, 3.2], P = 0.63).

### Effect of MiP on infant Hb levels during the first year of life

**Fig 3:**
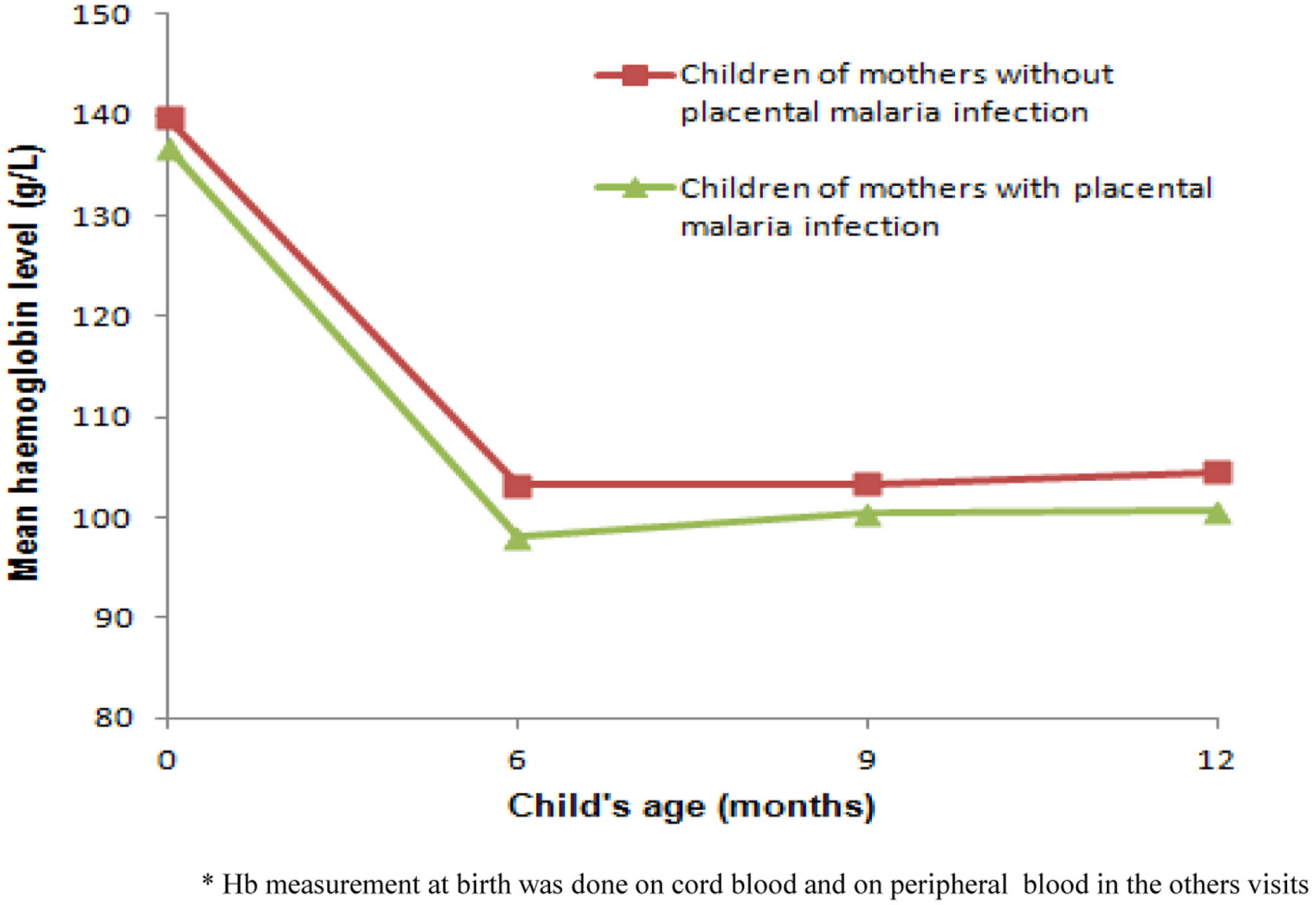
Changes of mean haemoglobin level of children during the first year of life according to mother's malaria status at delivery. Children born to mothers infected by malaria had a lower haemoglobin concentration than children born to non-infected mothers and this trend persisted during all first year of life.

Fig 3 shows infants' Hb concentrations variations during the follow-up according to the mothers' malaria status at delivery. Children born to mothers infected by malaria had a lower Hb concentration than children born to non-infected mothers and this trend persisted throughout the first year of life. With regard to the effect of MiP timing, only malaria infections occurring at delivery were significantly associated with a in infant Hb (dm = −4.7 g/L, 95% CI [-8.3, -1.1], P = 0.01). Malaria infections at 1^st^ and 2^nd^ ANC were not associated with infant Hb modification (Table 4).

Multilevel linear regression (Table 5) showed that maternal peripheral parasitaemia at delivery and placental malaria infection were the main maternal factors to negatively impact on infant Hb concentrations during the first year of life (dm = −4.6 g/L, 95% CI [-7.9, -1.3], P = 0.007; dm = −2.8 g/L, 95% CI [-5.3, -0.3], P = 0.03; respectively). Low pre-pregnancy BMI, specifically a severe thinness, was associated with a decrease in Hb concentrations during infancy (dm = −14.8 g/L, 95% CI [-25.9, -3.9], P = 0.05). Others factors to be significantly associated with a decrease in infant Hb concentrations during the first year of life were the occurrence of malaria infection during infancy (dm = −11.9 g/L, 95% CI [-14.7, -9.1], P < 0.01), inflammatory syndrome (dm = −3.9 g/L, 95% CI [-5.8, -1.9], P < 0.01) and fever (dm = −4.1 g/L, 95% CI [-6.5, -1.7], P < 0.01) at the time of Hb measurement. The number of maternal anaemia episodes and LBW were not associated with infant Hb during the first year of life (dm = −0.5 g/L, 95% CI [-1.6, 0.6], P = 0.37; dm = −3.1 g/L, 95% CI [-7.5, 1.2], P = 0.16, respectively).

**Table 5:** Relation between placental malaria infection or maternal peripheral parasitaemia at delivery and infant haemoglobin level (g/L) during the first year of life in district of Allada, Benin 2010–2012, N = 337 (multilevel linear regression).

Factors			Adjusted^†^ mean Hb difference (g/L)	95% CI	*P value*
Fixed effects*					
Maternal peripheral parasitaemia at delivery	Reference	No			
		Yes	- 4.6	[-7.9, -1.3]	0.007
Placental malaria infection	Reference	No			
		Yes	- 2.8	[-5.3, -0.3]	0.03
Random effects**					
Child-to-child variation (π_00_)			6.4	[5.3, 7.7]	-
Residual variation (σ²)			11.8	[11.1, 12.5]	-

Hb: Haemoglobin; 95% CI: Confidence Interval to 95%

^†^ Adjusted for estimation of pre-pregnancy body max index, infant malaria infection, fever episode and inflammatory syndrome, acid folic concentration at birth and infant age

* Estimated by maximum likelihood method

** Estimated by restricted maximum likelihood method.

The intraclass coefficient of Hb variations was estimated at 0.35. Thus, 65% of the total variance could be explained by the model.

### Population-attributable risks

A total of 11.8% (95% CI = 10.1–13.5%), 9.2% (95% CI = 7.6–10.7%), 7.7% (95% CI = 6.3–9.1%), 29.8% (95% CI = 27.3–32.3%) of infant anaemia in this population were attributable to maternal peripheral parasitaemia at delivery, placental malaria infection, maternal underweight and malaria infection during infancy, respectively.

## Discussion

This study has showed an important reduction of Hb concentrations in Beninese infants born to mothers with peripheral parasitaemia at delivery or placental malaria infection compared to infants from uninfected mothers. Our study also found a high prevalence of anaemia throughout infancy in the study population.

To our knowledge, this is one of the few studies to evaluate the effect of MiP on infant Hb variations through a one-year follow-up of the newborns in Sub-Saharan Africa (SSA). As Hb varies physiologically during the first year of life, an important strength of our study was to use a multilevel linear regression, best adapted to repeated measures to take into account the effect of time on infants’ Hb levels. Our analyses suggested that 65% of the total variance could be explained by the mixed model. Therefore, this model seems to be well suitable to the analyses of data. We also recorded few missing data (1.2%) reflecting the good quality of the follow-up. Furthermore, we were able to take into account potential confounding factors like estimated pre-pregnancy BMI, infants’ malaria infections and nutritional status (iron, folic acid, vitamin B12) at different times during the follow-up period.

We found that infant’s anaemia was very prevalent in southern Benin, particularly in the second half of the first year of life (> 65%). These high proportions are consistent with data from the Beninese Departmental Health and Demographic survey [21]. In our study, the Hb concentration decreased from birth to 6 months and then remained almost constant until 12 months. This decrease is known as the “physiologic anaemia of the newborn”. After Hb has reached its lowest level at ~2 months, it slowly increases again and becomes more or less stable between 6 and 9 months [22].

When we considered the effect of MiP timing, only maternal peripheral parasitaemia at delivery was significantly associated with decreasing of infant Hb. In our study, malaria infection decreased from 1^st^ to 2^nd^ ANC probably because of IPTp efficacy in parasite clearing [23], therefore the impact of malaria occurring during this period may be reduced by IPTp administration. The negative effect of maternal peripheral parasitaemia at delivery on infant Hb may also be explained by the timeframe of IPTp administration. Indeed the duration between the 2^nd^ IPTp dose and delivery had frequently exceeded two months. Consequently the pregnant women remained insufficiently protected for the last weeks of pregnancy, after the second IPTp dose. A similar result had previously been reported on Beninese pregnant women, clearly demonstrating that women who had an early intake of last IPTp dose were more at risk of LBW [24].

Maternal peripheral parasitaemia at delivery and placental malaria infection were the main maternal factors that contributed to reduce infant Hb concentrations during the first year of life. Placental malaria is one of the major features of malaria during pregnancy and it has been widely used as a standard indicator to characterize malaria infection in epidemiological investigations [25,26]. Moreover, placental histology used to assess MiP is considered as the "gold standard" of malaria diagnosis in pregnancy for epidemiological or biological study purposes, because it can show signs of active, chronic or past infections [13,27].

The biological explanation of the effect of MiP on infant anaemia in this study is complex to establish. Infant anaemia may be indirectly due to a higher susceptibility to malaria, a well-known risk factor for anaemia in itself. Two potential mechanisms can be evoked. First, environmental factors are likely to play a role, as women infected by malaria at delivery may have been more exposed to anopheles bites during pregnancy than uninfected women. Because mothers infected by malaria parasites and their children live in the same area, the latter may also be more exposed to the risk of malaria, one of the most important causes of infants' anaemia. Secondly, an immune tolerance phenomenon can also be involved, a very likely mechanism, as in the analysis of our dataset the relationship between MiP and decrease of infant's Hb persisted after adjusting on infant malaria infection throughout the first year of life. In a recent study on a similar population in Benin, Le Port *et al*. showed that first malaria infections in early childhood could be attributed independently to both placental malaria and high levels of exposure to infected anopheles [9]. In the same area, Rachas *et al*. showed that placental malaria infection was strongly associated with an increased risk of non-malaria fever episodes as well as gastrointestinal and respiratory febrile syndromes [28]. It can be assumed that the sequestration of malaria parasites in the placenta allows *in-utero* exposure of the fetus to a variety of malaria antigens that may induce immunological tolerance [29] and thus increase the susceptibility of infants to subsequent malaria infections [30]. Such immune tolerance could be responsible for the development of a tolerogenic environment, involving a number of immune effectors [31,32] potentially interacting with the development of immunity during the first year of life. Such interactions concern not only the malaria-specific immune response but also general immunity.

Earlier reports from Malawi and Cameroon had previously suggested a relation between placental malaria and infant Hb status. Redd *et al*. (1994) in Malawi had assumed that placental malaria infection might interfere with the hematologic status of the infant independently of birth weight and prematurity [33]. Cornet *et al*. (1998) had also reported that placental malaria infection was the strongest risk factor for anaemia in six-month-old children in southern Cameroon [34]. These studies have also suggested that physical alterations of the trophoblastic membrane due to malaria infection could decrease nutritional exchanges, such as iron, folate, and vitamin B12, between the mother and the fetus [35].

Our study suggests that children born to mothers with a very low BMI at the beginning of pregnancy (< 16 kg/m²) had a lower Hb concentration than children born to mothers with a normal BMI. It is also the first time that this relation is described. The low BMI reflects the maternal under-nutrition and could be a proxy of future infant's nutrition. Indeed, children born to underweight mothers may have a poor diet and therefore may be at higher risk of anaemia. Furthermore, underweight and stunted women are at high risk of delivering premature or LBW infants, who in turn, are at high risk of poor growth and anaemia in childhood and adolescence [36]. Indeed in our study, infants born to mothers with a BMI < 20 kg/m² had a 2.1 fold increased risk of LBW (95% CI [1.04–4.19], p = 0.04).

The number of maternal anaemia episodes during pregnancy was not associated with infant Hb concentration at birth and during the first year of life. It may seem surprising but similar results have been reported in Malawi [37], which may partially be explained by the fetal regulation of iron transport during pregnancy. A hierarchy in the use of iron in pregnancy has been suggested: fetus has priority over maternal iron stores [38]. An infant may be born with adequate iron stores though the mother stays moderately anemic during pregnancy. But according to Gambling *et al*., this mechanism also depends on the intensity of maternal iron depletion (below 200 μg/g, the maternal liver responds to the iron deficiency and tries to restore its own concentrations of iron). In our study, we have only considered the number of anaemia episodes detected at IPTp administrations and this may not necessarily reflect the history of maternal anaemia during all pregnancy duration. Indeed, by studying repeated measures of Hb on a larger sample size (1005 mother-infant pairs) and by using a path analysis, Ouédraogo *et al*. found a direct, though border-line, association of maternal anaemia in the third trimester and newborn's hemoglobin level at birth (personal communication).

Malaria infection, fever and inflammatory syndrome in children at the time of Hb measurement were significantly associated with a decrease in infant Hb concentrations probably due to hemolytic anaemia. The association of anaemia with fever or inflammatory syndrome underlines the important contribution of infections in the occurrence of infant anaemia during the first months of life and the potential causes of inflammation in pregnancy as well as in infancy hence deserve further studies.

This study has some limitations that should be considered. First, the absence of entomological data in our study such as the level of anopheles transmission did not allow the determination of the part of malaria risk attributable to a common exposure of the mother and her child to malaria vectors, even if the results rather suggest an effect of placental parasite sequestration on the immune tolerance of child. Second, the maternal nutritional status was assessed by BMI and weight was calculated approximately on the assumption of a 1kg/month gain from the end of the first trimester, which may not reflect correctly the nutritional status of women at the beginning of pregnancy because of the difference of weight gain from underweight to obese [39]. Further studies actually taking into account the nutritional status before pregnancy seem necessary to confirm our result on the relation between lower maternal BMI and the infant Hb during the first year of life. And finally, fundal height measurement by bimanual palpation was probably not the most suitable and accurate way to evaluate gestational age during pregnancy and may be the source of some imprecision in our final results.

## Conclusion

Anaemia in infancy is very common in Benin. In the present study, MiP and malaria infection during infancy were independently associated with a substantial reduction in infant Hb during the first year of life. Low maternal BMI at the beginning of pregnancy was also related to a decrease in infants' Hb. Strategies to prevent anaemia in infants should as well integrate measures during pregnancy such as the prevention of MiP and the improvement of maternal nutritional status. IPTp and the use of bed nets by both mothers and infants should be reinforced. Micronutrient supplementation should also be implemented in women of childbearing age and pregnant women but with a lot of caution in a context of malaria-iron interactions where the protection of iron deficiency against malaria is still a controversial issue [40,41].

## Supporting Information

S1 Fig Haemoglobin variation in Beninese infants during the fisrt year of life. During the first months of life, haemoglobin level declines from very high level at birth to its lowest level at 2–3 months of age. This decrease is known as the “physiologic anaemia of the newborn”. After Hb has reached its lowest level at ~2 months, it slowly increases again and becomes more or less stable between 6 and 9 months.
(TIF)
