# Supplementary figures and images for: Malaria in Pregnancy Is a Predictor of Infant Haemoglobin Concentrations during the First Year of Life in Benin, West Africa
Source: PLoS One. 2015 Jun 8;10(6):e0129510. doi: 10.1371/journal.pone.0129510 (PMC4460073; doi:10.1371/journal.pone.0129510)

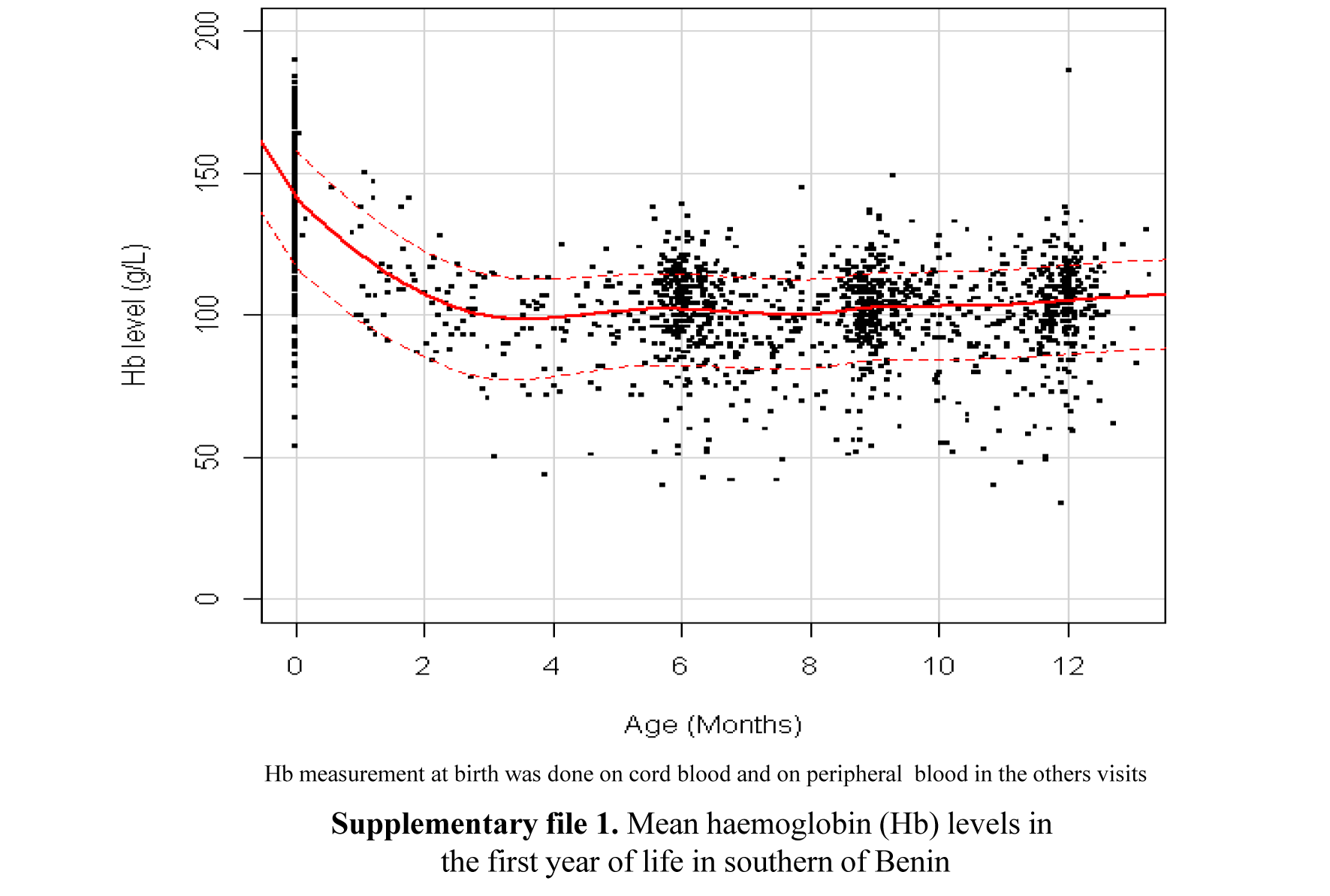

Supplement: S1 Fig — During the first months of life, haemoglobin level declines from very high level at birth to its lowest level at 2–3 months of age. This decrease is known as the “physiologic anaemia of the newborn”. After Hb has reached its lowest level at ~2 months, it slowly increases again and becomes more or less stable between 6 and 9 months. (TIF) [file pone.0129510.s001.tif]
